# The Early Awareness and Alert System in Sweden: History and Current Status

**DOI:** 10.3389/fphar.2017.00674

**Published:** 2017-10-05

**Authors:** Irene Eriksson, Björn Wettermark, Marie Persson, Morgan Edström, Brian Godman, Anna Lindhé, Rickard E. Malmström, Helena Ramström, Mia von Euler, Anna Bergkvist Christensen

**Affiliations:** ^1^Department of Healthcare Development, Stockholm County Council, Stockholm, Sweden; ^2^Department of Medicine Solna, Karolinska Institutet, Solna, Sweden; ^3^Healthcare Administration, Stockholm County Council, Stockholm, Sweden; ^4^Department of Clinical Pharmacology, County Council of Östergötland, Linköping University Hospital, Linköping, Sweden; ^5^Health Economics Unit, University of Liverpool Management School, Liverpool, United Kingdom; ^6^Clinical Pharmacology, Karolinska University Hospital, Stockholm, Sweden; ^7^Strathclyde Institute of Pharmacy and Biomedical Sciences, University of Strathclyde, Glasgow, Scotland, United Kingdom; ^8^Department of Healthcare, Regional Head Office, Region Västra Götaland, Gothenburg, Sweden; ^9^Department of Clinical Science and Education, Södersjukhuset, Karolinska Institutet, Solna, Sweden; ^10^Department of Medicines Management and Informatics, Regional Head Office, Region Skåne, Malmö, Sweden

**Keywords:** early awareness and alert systems, horizon scanning, health technology assessment, innovation, health policy, Sweden

## Abstract

**Introduction:** Over the past decades, early awareness and alert (EAA) activities and systems have gained importance and become a key early health technology assessment (HTA) tool. While a pioneer in HTA, Sweden had no national level EAA activities until 2010. We describe the evolution and current status of the Swedish EAA System.

**Methods:** This was a historical analysis based on the knowledge and experience of the authors supplemented by a targeted review of published and gray literature as well as documents relating to EAA activities in Sweden. Key milestones and a description of the current state of the Swedish EAA System is presented.

**Results:** Initiatives to establish a system for the identification and assessment of emerging health technologies in Sweden date back to the 1980s. In the 1990s, the Swedish Agency for HTA and Assessment of Social Services (SBU) supported the development of EuroScan as one of its founder members. In the mid-2000s, an independent regional initiative, driven by the Stockholm County Drug and Therapeutics Committee, resulted in the establishment of a regional horizon scanning function. By 2009, this work had expanded to a collaboration between the four biggest counties in Sweden. The following year it was further expanded to the national level and since then the Swedish EAA System has been carrying out identification, filtration and prioritization of new medicines, early assessment of the prioritized medicines, and dissemination of information. In 2015, the EAA System was incorporated into the Swedish national process for managed introduction and follow-up of new medicines. Outputs from the EAA System are now used to select new medicines for inclusion in this process.

**Conclusions:** The Swedish EAA System started as a regional initiative and rapidly grew to become a national level activity. An important feature of the system today is its complete integration into the national process for managed introduction and follow-up of new medicines. The system will continue to evolve as a response both to the changing landscape of health innovations and to new policy initiatives at the regional, national and international level.

## Introduction

The Health Technology Assessment (HTA) Glossary defines horizon scanning as the systematic identification of health technologies that are new, emerging or becoming obsolete and that have the potential to effect health, health services and/or society (HTA, 2017a). Such systematic identification is typically carried out as one element within a wider range of related activities, which is why the term “early awareness and alert system” has been proposed to capture the extent of the work immediately following horizon scanning (Packer et al., 2012). An early awareness and alert (EAA) system is a system that aims to identify, filter and prioritize new and emerging health technologies, or new uses of existing interventions; to assess or predict their impact on health, health services and/or society; and to disseminate information (HTA, 2017b). The extent of how information about new and emerging medicines is used by decision makers can differ, but the consensus is that timely identification and assessment is generally helpful in supporting the adoption and use of new health technologies to the benefit of patients (WHO, 2015).

Over the past two decades, EAA activities and systems have gained importance and become a key early HTA tool (Wettermark et al., 2010a; Godman et al., 2015; WHO, 2015). Many of the existing EAA systems are part of the International Information Network on New and Emerging Health Technologies (EuroScan International Network) that currently includes 18 member agencies representing a number of European countries as well as Australia, New Zealand, Canada, South Korea and Israel (EuroScan, 2017). There are also EAA initiatives outside the EuroScan International Network, such as the Agency for Healthcare Research and Quality (AHRQ) Healthcare Horizon Scanning System in the United States (AHRQ, 2017), the Specialist Pharmacy Service (SPS) in the United Kingdom (NHS, 2017), and the Swedish EAA System.

While much has been written about the member agencies of the EuroScan International Network (Simpson et al., 2008; Packer et al., 2012, 2015), to date there has been no description of the Swedish EAA System published in the literature, although its predecessor was mentioned first in an article on a model for structured introduction of new medicines published in Swedish (Gustafsson et al., 2008) and then in an article on the forecasting model developed and used by a regional payer in Sweden (the Stockholm County Council) (Wettermark et al., 2010b). This is an important gap as the Swedish EAA System is part of a comprehensive approach to optimize the introduction of new medicines (The Swedish Association of Local Authorities and Regions, 2017a). To address this gap, we documented the development of the Swedish EAA System and provided a description of its current status. We believe this will be of interest to other countries facing similar challenges with the introduction of new premium priced medicines alongside aging populations and fixed finite budgets.

## Methods

This overview of the Swedish EAA System is based on the knowledge and experience of the authors supplemented by a targeted review of published and gray literature as well as documents relating to EAA activities in Sweden.

The areas explored in our historical analysis included the early development of EAA activities in Sweden, the work of the Swedish Agency for HTA and Assessment of Social Services (SBU) around early identification and assessment of new technologies, regional horizon scanning initiatives and the process of their expansion to the national level as well as the integration of regional initiatives into the national process for the structured introduction and follow-up of new medicines.

In the documentation of the early development of EAA activities in Sweden and the work of SBU, we mainly relied on a targeted literature review. Relevant papers on EAA activities were identified on PubMed using the terms “horizon scanning,” “early warning system” and “early awareness and alert” combined with “medicine,” “medication” and “drug.” A manual check of references for relevant articles was then conducted to identify further papers. The following websites of the key actors were also searched: Janusinfo (the website of the Drug and Therapeutic Committee and the Health and Medical Care Administration of the Stockholm County Council) (Janusinfo, 2017), the Swedish Association of Local Authorities and Regions (SALAR) (The Swedish Association of Local Authorities and Regions, 2017b), the EuroScan International Network (EuroScan, 2017), and SBU (The Swedish Agency for HTA and Assessment of Social and Services, 2017). Finally, additional relevant publications known to the authors were reviewed.

In the documentation of the regional horizon scanning initiatives, and the process of their expansion to the national level, as well as the integration into the national process for the structured introduction and follow-up of new medicines, we relied on the knowledge and experience of the authors.

### Setting

The key aim of Swedish health policy, similar to that of other countries with universal healthcare coverage, is to provide equitable and comprehensive healthcare for its citizens. Healthcare in Sweden is primarily funded through direct taxation (Anell et al., 2012; Anell, 2015). Overall health policy in Sweden is governed at the national level with, for example, the Dental and Pharmaceutical Benefits Agency (TLV) maintaining responsibility related to value-based pricing and reimbursement of outpatient prescription medicines (Dental and Pharmaceutical Benefits Agency, 2017). However, decision making is to a large extent decentralized to county councils responsible for providing healthcare and medicines to their residents (Anell et al., 2012). Regional Drug and Therapeutic Committees (DTCs) play an important role in facilitating rational use of medicines at the regional level (Godman et al., 2009). All inpatient medicines are paid for in full by the county councils. For reimbursed prescription medicines, the county councils receive a government grant that is negotiated between the national government and SALAR.

**Figure 1:**
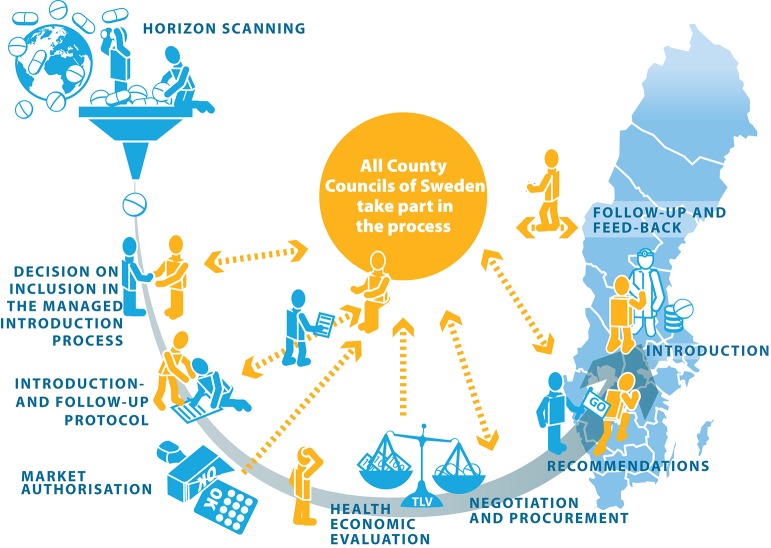
The Swedish national process for managed introduction and follow-up of new medicines. Source: The Swedish Association of Local Authorities and Regions (2017a). Reproduced with permission from Sofie Alverlind.

Over the past decade, there has been continuous development of processes for the structured introduction and follow-up of new medicines both at the regional and national level. In 2006, the Stockholm County Council established a regional model for the introduction and rational use of new medicines (Gustafsson et al., 2008). As a central part of the National Pharmaceutical Strategy introduced in 2011, SALAR has led initiatives with the aim to develop a national collaboration to promote more effective and safer use of new medicines (Medical Products Agency, 2017). Today, a national collaboration model is in place for the managed introduction and follow-up of new medicines that brings together the county councils, DTCs, governmental agencies and that also facilitates interaction with pharmaceutical companies (see Figure 1). Key stakeholders in this model include the county councils, TLV, and the newly formed New Therapies (NT) Council that is commissioned to make recommendations on the use of new medicines with the aim of enabling equal treatment for patients throughout the country (The Swedish Association of Local Authorities and Regions, 2017a). The NT Council is made up of regional representatives with medical or pharmaceutical expertise. In addition, the NT Council has members with expertise in other areas such as ethics and health economics.

## Results

We first provide an overview of the evolution of EAA activities in Sweden, including key milestones, and then describe the current status of the Swedish EAA System.

### Development of EAA activities in sweden

Initiatives to establish a system for the identification and assessment of emerging health technologies in Sweden date back to the 1980s. In 1984, the Center for Medical Technology Assessment was established at Linköping University in collaboration with the local regional payer (i.e., the county council). A few years later, SBU was established and by the mid-1990s, a pilot project had been initiated with the objective to develop and test the feasibility of a model for a national system for early identification and assessment of new technologies (Carlsson et al., 1998). This project laid the groundwork for establishing SBU Alert in 1997. SBU also actively supported the development of EuroScan and was one of its founder members (Carlsson et al., 1998; Simpson et al., 2008). SBU however never assumed a function of identifying new and emerging health technologies, but rather became an HTA body carrying out assessments of both new and established technologies (diagnostics, prevention, treatments) based on proposals coming from various stakeholders, including clinicians, county councils and decision making authorities, such as the Medical Products Agency (MPA) and TLV (Packer et al., 2015). In parallel, the regional DTCs were tracking new and emerging medicines, thus providing decision makers with information, albeit not in a structured and systematic way. Consequently, a structured EAA system suitable for the needs of the entire healthcare system (including healthcare payers, clinicians, and other stakeholders) had to be developed and, by 2007, the Stockholm County Council had established a horizon scanning function (Gustafsson et al., 2008).

The creation of this horizon scanning function was driven by the Stockholm County DTC that, just like other regional DTCs, had been successful in facilitating rational use of existing medicines, particularly in general practice (Wettermark et al., 2008; Godman et al., 2009; Gustafsson et al., 2011; Kardakis et al., 2015). During the 2000s, the profile of pharmaceutical innovation however was markedly changing and many more medicines in the development pipeline were intended for use in specialized care. It was recognized that a proactive approach with regards to new specialist medicines was warranted. Uncertainty around patient outcomes was a key driver in this initiative, however the growing budget impact of new specialist medicines was also a contributing factor (Wettermark et al., 2010b). The Stockholm County Council's horizon scanning function enabled better identification and prioritization of new medicines, particularly new medicines likely to be used in specialized care. This function was largely influenced by the United Kingdom's National Institute for Health Research Horizon Scanning Research and Intelligence Centre (NIHR HSRIC), which also provided advice and support in its establishment.

By 2009, the work of the Stockholm County Council's horizon scanning function had expanded to a collaboration between the four biggest county councils in Sweden. This collaboration on horizon scanning became of interest to other county councils too, which resulted in expanding the EAA activities to a national level collaboration facilitated by SALAR. Since 2010, a working group consisting of staff from the four biggest county councils has been carrying out EAA activities on behalf of all counties in Sweden. In 2015, these EAA activities were incorporated into the Swedish national process for managed introduction and follow-up of new medicines, which integrated the EAA activities as the first step of this process.

### Description of the Swedish EAA system

In Sweden, the national level EAA activities are referred to as horizon scanning. In this paper, we will refer to these activities as the Swedish EAA System, even though this has not been adopted as its official name, as the scope of the work conducted by the system is most accurately reflected by this term.

#### Objective and scope of the Swedish EAA system

The aim of the Swedish EAA System is to support the long-term planning of the county councils and to optimize the readiness of the healthcare system to the introduction of new medicines. The working group continuously identifies new medicines and indications expected to be granted marketing authorization within the next 1–3 years.

#### Organization and resources

The Swedish EAA activities are publicly funded. A working group coordinator leads the work together with a small team consisting of pharmacists. To gauge the potential impact that new identified medicines will have on the healthcare system, the group works closely with the regional DTCs. Affiliated experts such as clinical pharmacologists and clinicians provide additional guidance, for example by assessing unmet medical needs and helping to estimate the value of a new medicine compared to already existing treatment options. The responsibilities of the working group include identification, filtration and prioritization, early assessment, and dissemination of results.

#### Customers

The main users of the outputs of the Swedish EAA System are the county councils (the DTCs and strategic functions) and the NT Council. The outputs include a horizon scanning database of identified new and emerging medicines, a list of prioritized medicines, individual early assessment reports on prioritized medicines and indications, and quarterly newsletters. Based on the information from the early assessment reports, the NT Council makes a decision on whether the medicine should be included in the national process for managed introduction and follow-up.

#### Methods used in identification, filtration, and prioritization

##### Identification

This initial step covers identifying new and emerging medicines and new therapeutic indications of existing medicines. The identification process generally spans medicines from phase II–III of the development until regulatory approval. Medicines estimated to be of high impact and therefore likely to gain accelerated assessment with the European Medicines Agency (EMA), such as certain cancer or orphan medicines, are included from phase II.

A structured search methodology is employed that includes a variety of sources that can be classified as primary (e.g., the manufacturer), secondary (e.g., knowledge or expertise intended for other purposes) and tertiary (e.g., other EAA systems' efforts to identify technologies) (Robert et al., 1998). Primary sources comprise yearly pipeline meetings with individual pharmaceutical companies. Additional information is obtained as needed through company contacts and supplemented with searches of company websites, including press releases and investor reports. Secondary sources of information include regulatory agencies, scientific journals, conference proceedings, and health technology media outlets. Examples of secondary sources used include the Swedish MPA, EMA, the United States Food and Drug Administration, pharmaceutical industry news sources (e.g., First Word Pharma) and registries of clinical studies such as ClinicalTrials.gov. Furthermore, a note is taken of medicines identified by other EAA systems, such as the NIHR Innovation Observatory and the SPS. The scanning frequency of these secondary and tertiary data sources vary from daily to monthly. All identified medicines are entered into a horizon scanning database that is accessible to all working group members and representatives from the county councils.

##### Filtration and prioritization

The filtration and prioritization step involves the evaluation of which identified medicines may be of importance and impact treatment of patients or the healthcare system. Medicines with an expected marketing authorization date in the coming few years are screened using the following filtration criteria: the patient population size; burden of disease; budget impact; anticipated clinical benefits; level of innovation; organizational impact; impact on treatment guidelines; safety aspects; level of interest from media and patient organizations; anticipated sub-optimal market uptake; and relevance from a legal, ethical and/or political aspect (see the list in Box 1) (The Swedish Association of Local Authorities and Regions, 2017c). The filtration and prioritization activities follow a quarterly schedule.

Box 1 **Filtration criteria.**

Size of patient population

Severity of the disease

Potential to clinically improve patient outcomes

Innovative way of treating the disease

Potential to affect treatment costs

May require reorganization of the healthcare system

Potential to influence treatment guidelines

Potential safety issues

Potentially high media/public interest

Non-optimal introduction rate following marketing authorization

Potentially legally, ethically, or politically interesting

It is also taken into consideration whether the medicine/indication:

Belongs to a growing class of medicines or therapeutic area

Represents a new form of treatment or a new class of medicines

Is revelant to Swedish conditions

Is in late phase clinical trials (Phase II or Phase III) or is submitted to regulatory agencies

In an initial filtration step, the group identifies medicines that may have a potential impact on the healthcare system based on the filtration criteria and summarizes these medicines by therapeutic area. The results are distributed via the working group to experts affiliated with the regional DTCs and, if the necessary expertise is not available through the DTCs, to other experts for a comment on the new medicine. The experts then prepare a brief assessment summary. These summaries focus on the following key aspects: level of innovation; unmet medical need; patient population; severity of the disease; potential safety aspects; media interest; budget impact; and need for changes to the structure of healthcare delivery should the medicine be approved for use.

As a second filtration step, the working group meets to discuss and prioritize (using the same criteria as in the initial filtration step) the identified medicines based on the brief assessment summaries. After the meeting, the identified and prioritized medicines are finally put on a short list and flagged for subsequent assessment. This list of prioritized medicines also contains separate sections for completed and ongoing assessments as well as orphan medicines that have been submitted to EMA for marketing authorization. The prioritization list is subsequently shared within the national collaboration for managed introduction and follow-up of new medicines. An early assessment report is produced for each prioritized medicine.

#### Outputs

**Figure 2:**
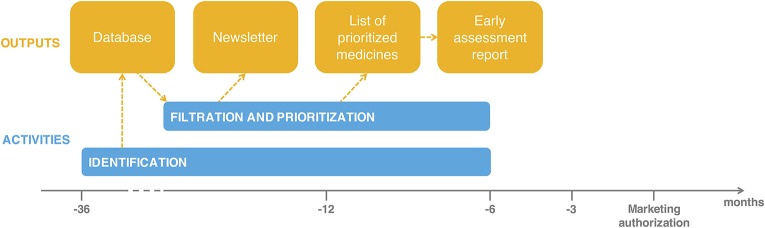
Activities and outputs of the Swedish EAA System.

The main deliverables of the Swedish EAA System include the horizon scanning database of identified new medicines, the list of prioritized medicines, individual early assessment reports on prioritized medicines and indications, as well as quarterly newsletters (see Figure 2).

##### Horizon scanning database of identified new medicines

The horizon scanning database comprising all new and emerging medicines identified is accessible to all working group members and is also provided to representatives from the county councils. Information collected in this database includes the chemical or code name in the early stages of development as well as the common/brand name and the Anatomical Therapeutic Classification (ATC) code. Additional basic information includes the therapeutic area, indication, mechanism of action and the manufacturer/developer. Furthermore, the group also documents development status (including predicted and actual marketing authorization submission date and EMA filing status), the predicted marketing authorization date and whether the identified medicine or indication has an orphan medicine status designation.

##### List of prioritized medicines

The updated list of prioritized medicines with information on the completed, ongoing and planned assessments is also made available to the customers following the filtration and prioritization step.

##### Early assessment report

The work on an early assessment report is initiated following the manufacturer's submission of the application for marketing authorization to EMA. Upon confirmation of filing with the regulatory authorities, the working group coordinator informs the working group member tasked to write that specific report. This working group member then contacts an assigned expert, most often a clinical pharmacologist or in some cases another clinician, who initiates the writing process. The four county councils take turns in writing the reports. Approximately 20 early assessment reports are produced per year.

The completed draft assessment report is shared within the working group for further input. The three other county council representatives facilitate an independent expert review of the assessment report within their respective county. All comments are then collated and implemented by the working group coordinator to produce the final early assessment report.

The early assessment report is intended to provide a summary of the potential value and impact on healthcare delivery that an upcoming medicine or new indication would bring. The report follows a structured template and is based on early information prior to marketing authorization (see Box 2). The important requirement for the report is that the information used must come from publicly accessible sources. As such, it does not provide a formal assessment of a medicine but rather gives the regional decision makers early information ahead of the availability of a new medicine. The report describes the medicine's mechanism of action, summarizes what is known about efficacy and safety from the pivotal trials, and provides an overview of the therapeutic area, existing treatment guidelines and how the new medicine would impact healthcare delivery from an economic and organizational standpoint. In addition, the report may provide an overview of how the introduction and uptake of the new medicine can be followed up and assessed in clinical practice, for example with the use of existing healthcare quality registries.

Box 2 **Components of the early assessment report.**

Expert comment/opinion

Description of the medicine

Estimated time to approval

Clinical need and size of patient population

Who will prescribe the medicine

Current treatment alternatives

Clinical efficacy

Clinical observations/safety

Completed and ongoing studies

Other relevant medicines in the pipeline

Estimated cost

Clinical, service, and financial impact

Possibility to monitor utilization post-launch

Other markets

Sales arguments

The report's conclusions are not updated when new information becomes available; however, regulatory information such as filing and reimbursement status is appended when available.

##### Newsletter

The working group publishes a newsletter four times per year following the filtration and prioritization step. The newsletter is distributed to members of the national collaboration for managed introduction and follow-up of new medicines. It reports on newly identified medicines and indications, recently published assessment reports, and which medicines were selected for assessment during the most recent meeting. The newsletter also contains updates from EMA, MPA, and TLV that are of relevance from a strategic planning perspective.

#### Expert involvement

As mentioned, experts are engaged during the filtration, prioritization and early assessment steps. The input from these experts is central in the development of the early assessment report. The working group members from the four counties are responsible for putting together the relevant expertise as needed, primarily relying on experts affiliated with the regional DTCs. All experts involved in the early assessment of new medicines are asked to declare any conflicts of interest.

## Discussion

In this paper, we provided an overview of the evolution of the Swedish EAA System from the early discussions in the 1980s to the recent regional work that was expanded to include other regions and eventually integrated into the national process for managed introduction and follow-up of new medicines. Furthermore, we described the current state of the Swedish EAA System. Such EAA activities and systems have been developed in many other countries during the last decades (Packer et al., 2015; WHO, 2015). In their efforts to facilitate rational and cost-effective use of medicines, healthcare administrators have broadened their pharmaceutical policy focus from reforms to promote the use of generics and facilitate the rational use of existing medicines to the establishment of new models that improve the introduction of new medicines (Godman et al., 2015; Matusewicz et al., 2015).

The Swedish EAA System has a lot in common with established EAA activities and systems. Its objective and filtration and prioritization criteria are similar to that of the EuroScan International Network members (Packer et al., 2015). The most obvious differences lie in the type of technology covered, the time when the identification begins, the customer base and the degree of integration with other processes.

The Swedish EAA System focuses only on medicines, while agencies in other countries also cover devices, diagnostics, interventional procedures and structural changes in healthcare delivery and organization (Packer et al., 2015). The focus on medicines may be explained by the track record and knowledge of both the founding Stockholm County DTC and the DTCs of the other collaborating counties with a strong base in the clinic and academia, including experts from pharmacy, clinical pharmacology and clinicians from different therapeutic areas (Godman et al., 2009). Furthermore, the considerable impact of medicines on healthcare budgets (the number two driver of costs after staff costs) reinforced that medicines became the health technology covered by the Swedish EAA System (Godman et al., 2009). It is possible that the focus will change in the future as the Swedish county councils also are responsible for almost all other parts of healthcare delivery. Consequently, there is a clear potential to leverage the work around the introduction of new medicines to also get a more rational introduction of other health technologies.

The identification of new medicines starts at various time points for different agencies (Lepage-Nefkens et al., 2017). For example, the Italian Horizon Scanning Project issues reports as early as 36 months prior to expected EMA authorization (Joppi et al., 2009). The Scottish Medicines Consortium provides information on all new medicines expected to reach the market within the next calendar year (Scottish Medicines Consortium, 2015). The Swedish early awareness reports are typically published 6 months before the authorization of a new medicine. No evaluation has been made so far on the precision and appropriateness of the timing. Assessments that are produced too early may be of limited value for decision makers as they would be based on scarce published data with many of the evaluated medicines never reaching the market (Ermisch et al., 2016). On the other hand, reports published too late may not fulfill the need for preparatory activities, such as the set-up of registries to monitor the introduction of a new technology including its effectiveness and safety in routine clinical practice, the planning of budgets and the development of quality indicators (Campbell et al., 2015; WHO, 2015).

Existing EAA systems have a wide variety of customers, ranging from national governmental health departments and ministers to hospitals, insurance or reimbursement organizations, healthcare professionals, medical advisors and clinical experts (Packer et al., 2015). The Swedish EAA System has clear ownership by the county councils, which are responsible for financing healthcare to fulfill the need of all residents. Consequently, the outputs of the Swedish EAA System are important for the prioritization of how resources are used. Furthermore, the EAA System is fully integrated in other national and regional processes related to the introduction of new medicines. These include forecasting, priority setting, pricing and reimbursement, guideline development and observational studies to monitor the uptake of new medicines (Gustafsson et al., 2008; Wettermark et al., 2010a,b). It is also important to acknowledge that the regional ownership facilitates involvement of clinical experts, which both increases the value of the assessments and is an important component of the adoption of new medicines.

There are several challenges that the Swedish EAA System may face in the future. First, the pharmaceutical area is rapidly changing with new types of technologies emerging such as cell and gene therapy medicines. These also have to be taken into account when prioritizing and estimating the impact of new health technologies. Second, for medicines that target high unmet medical need, several schemes exist to facilitate the regulatory pathway that can lead to new medicines being approved based on phase II studies. Adaptive pathways for new medicines are also being proposed (Ermisch et al., 2016). As a result of this, it can be difficult to foresee when a pharmaceutical company aims to apply for marketing authorization and if this application will be based on phase II or phase III studies. Third, the Swedish national process for managed introduction and follow-up of new medicines, launched in 2015, is currently being evaluated by the Swedish Agency for Health and Care Services Analysis. The scope of the evaluation is to describe the benefit of the process and to see whether the patients are gaining improved access to cost-effective medicines. The report is due in 2017 and as a possible consequence thereof the Swedish EAA System may be altered.

To summarize, the Swedish EAA System started as a regional initiative and rapidly grew to become a national level activity. An important feature of the EAA System today is its complete integration into the national process for managed introduction and follow-up of new medicines as well as the strong collaboration with experts from the regional DTCs. The EAA System will continue to evolve as a response both to the changing landscape of health innovations and to new policy initiatives at the regional, national and international level.

## Author contributions

IE, BW, and ABC developed the concept of the paper and produced the first draft. All authors critically revised the manuscript for important intellectual content. All authors read and approved the final version of the manuscript.

### Conflict of interest statement

The authors declare that the research was conducted in the absence of any commercial or financial relationships that could be construed as a potential conflict of interest.

## References

[B1] AHRQ (2017). AHRQ Healthcare Horizon Scanning System. The Agency for Healthcare Research and Quality Available online at: http://effectivehealthcare.ahrq.gov/index.cfm/who-is-involved-in-the-effective-health-care-program1/ahrq-horizon-scanning-system/ (Accessed June 1, 2017).

[B2] Anell A. Glenngård A. H. Merkur S. M. (2012). Sweden: health system review. Health Syst. Transit. 14, 1–159. Available online at: http://www.euro.who.int/__data/assets/pdf_file/0008/164096/e96455.pdf 22894859

[B3] Anell A. (2015). The public–private pendulum—patient choice and equity in sweden. N. Engl. J. Med. 372, 1–4. 10.1056/NEJMp1411430 25551523

[B4] Campbell S. M. Godman B. Diogene E. Furst J. Gustafsson L. L. MacBride-Stewart S.. (2015). Quality indicators as a tool in improving the introduction of new medicines. Basic Clin. Pharmacol. Toxicol. 116, 146–157 10.1111/bcpt.12295 25052464

[B5] Carlsson P. Hultin H. Tornwall J. (1998). The early experiences of a national system for the identification and assessment of emerging health care technologies in Sweden. Int. J. Technol. Assess. Health Care 14, 687–694. 10.1017/S0266462300012009 9885459

[B6] Dental and Pharmaceutical Benefits Agency (2017). Dental and Pharmaceutical Benefits Agency. Available online at: www.tlv.se/In-English/in-english/ (Accessed June 1, 2017).

[B7] Ermisch M. Bucsics A. Vella Bonanno P. Arickx F. Bybau A. Bochenek T.. (2016). Payers' views of the changes arising through the possible adoption of adaptive pathways. Front. Pharmacol. 7:305. 10.3389/fphar.2016.00305 27733828 PMC5039228

[B8] EuroScan (2017). The EuroScan International Network. Available online at: https://www.euroscan.org/ (Accessed June 1, 2017).

[B9] Godman B. Malmstrom R. E. Diogene E. Gray A. Jayathissa S. Timoney A.. (2015). Are new models needed to optimize the utilization of new medicines to sustain healthcare systems? Expert Rev. Clin. Pharmacol. 8, 77–94. 10.1586/17512433.2015.990380 25487078

[B10] Godman B. Wettermark B. Hoffmann M. Andersson K. Haycox A. Gustafsson L. L. (2009). Multifaceted national and regional drug reforms and initiatives in ambulatory care in Sweden: global relevance. Expert Rev. Pharmacoecon. Outcomes Res. 9, 65–83. 10.1586/14737167.9.1.65 19371180

[B11] Gustafsson L. L. Wettermark B. Godman B. Andersen-Karlsson E. Bergman U. Hasselstrom J.. (2011). The ‘wise list’- a comprehensive concept to select, communicate and achieve adherence to recommendations of essential drugs in ambulatory care in Stockholm. Basic Clin. Pharmacol. Toxicol. 108, 224–233. 10.1111/j.1742-7843.2011.00682.x 21414143

[B12] Gustafsson L. L. Wettermark B. Kalin M. Korkmaz S. Persson M. E. Almkvist H.. (2008). A model for structured introduction of new drugs. The aim is to offer all patients appropriate treatment. Lakartidningen 105, 2917–2922. Available online at: http://www.lakartidningen.se/OldWebArticlePdf/1/10486/LKT0842s2917_2922.pdf 19025148

[B13] HTA (2017a). The Health Technology Assessment (HTA) Glossary. Horizon Scanning. Available online at: http://htaglossary.net/HomePage (Accessed June 1, 2017).

[B14] HTA (2017b). The Health Technology Assessment (HTA) Glossary. Early Awareness and Alert System. Available online at: http://htaglossary.net/HomePage (Accessed June 1, 2017).

[B15] Janusinfo (2017). Janusinfo (The Website of the Drug and Therapeutic Committee and the Health and Medical Care Administration of the Stockholm County Council). Available online at: http://www.janusinfo.se/ (Accessed June 1, 2017).

[B16] Joppi R. Dematte L. Menti A. M. Pase D. Poggiani C. Mezzalira L. (2009). The Italian horizon scanning project. Eur. J. Clin. Pharmacol. 65, 775–781. 10.1007/s00228-009-0666-z 19495735

[B17] Kardakis T. Tomson G. Wettermark B. Brommels M. Godman B. Bastholm-Rahmner P. (2015). The establishment and expansion of an innovative centre for rational pharmacotherapy–determinants and challenges. Int. J. Health Plan. Manage. 30, 14–30. 10.1002/hpm.2202 23785014

[B18] Lepage-Nefkens I. Douw K. Mantjes G. de Graaf G. Leroy R. Cleemput I. (2017). Horizon Scanning for Pharmaceuticals: Proposal for the BeNeLuxA Collaboration. Health Services Research (HSR). Brussel: Belgian Health Care Knowledge Centre (KCE). Report No.: D/2017/10.273/15.

[B19] Matusewicz W. Godman B. Pedersen H. B. Furst J. Gulbinovic J. Mack A.. (2015). Improving the managed introduction of new medicines: sharing experiences to aid authorities across Europe. Expert Rev. Pharmacoecon. Outcomes Res. 15, 755–758. 10.1586/14737167.2015.1085803 26368060

[B20] Medical Products Agency (2017). Medical Products Agency. The National Pharmaceutical Strategy 2017. Available online at: http://www.lakemedelsverket.se/overgripande/Om-Lakemedelsverket/Nationell-lakemedelsstrategi (Accessed June 1, 2017).

[B21] NHS (2017). The NHS Specialist Pharmacy Service. Available online at: https://www.sps.nhs.uk (Accessed June 1, 2017).

[B22] Packer C. Gutierrez-Ibarluzea I. Simpson S. (2012). The evolution of early awareness and alert methods and systems. Int. J. Technol. Assess. Health Care 28, 199–200. 10.1017/S0266462312000426 22980694

[B23] Packer C. Simpson S. de Almeida R. T. (2015). EuroScan international network member agencies: their structure, processes, and outputs. Int. J. Technol. Assess. Health Care 31, 78–85. 10.1017/S0266462315000100 26077793 PMC4505737

[B24] Robert G. Gabbay J. Stevens A. (1998). Which are the best information sources for identifying emerging health care technologies? An international Delphi survey. Int. J. Technol. Assess. Health Care 14, 636–643. 10.1017/S0266462300011946 9885453

[B25] Scottish Medicines Consortium (2015). Scottish Medicines Consortium. Guidance on Horizon Scanning Process 2015. Available online at: https://www.scottishmedicines.org.uk/About_SMC/What_we_do/Horizon_Scanning/Guidance_on_Horizon_Scanning (Accessed June 1, 2017).

[B26] Simpson S. Packer C. Carlsson P. Sanders J. M. Ibarluzea I. G. Fay A. F.. (2008). Early identification and assessment of new and emerging health technologies: actions, progress, and the future direction of an international collaboration–EuroScan. Int. J. Technol. Assess. Health Care 24, 518–525. 10.1017/S0266462308080689 18828949

[B27] The Swedish Agency for HTA and Assessment of Social Services (2017). The Swedish Agency for Health Technology Assessment and Assessment of Social Services (SBU). Available online at: http://www.sbu.se/ (Accessed June 1, 2017).

[B28] The Swedish Association of Local Authorities and Regions (2017a). Managed Introduction - This is How it Works. Available online at: http://www.janusinfo.se/Managed-introduction–this-is-how-it-works/ (Accessed June 1, 2017).

[B29] The Swedish Association of Local Authorities Regions (2017b). The Swedish Association of Local Authorities and Regions. Available online: https://skl.se/ (Accessed June 1, 2017).

[B30] The Swedish Association of Local Authorities and Regions (2017c). Managed Introduction – This Is How It Works/Horizon Scanning. Available online at: http://www.janusinfo.se/Managed-introduction–this-is-how-it-works/Horizon-scanning/ (Accessed June 1, 2017).

[B31] Wettermark B. Godman B. Andersson K. Gustafsson L. L. Haycox A. Bertele V. (2008). Recent national and regional drug reforms in Sweden: implications for pharmaceutical companies in Europe. Pharmacoeconomics 26, 537–550. 10.2165/00019053-200826070-00001 18563945

[B32] Wettermark B. Godman B. Eriksson C. van Ganse E. Garattini S. Joppi R. (2010a). Einführung neuer Arzneimittel in europäische Gesundheitssysteme. G+G Wissenschaft 10, 24–34. Available online at: https://www.wido.de/fileadmin/wido/downloads/pdf_ggw/wido_ggw_aufs3_0710.pdf

[B33] Wettermark B. Persson M. E. Wilking N. Kalin M. Korkmaz S. Hjemdahl P.. (2010b). Forecasting drug utilization and expenditure in a metropolitan health region. BMC Health Serv. Res. 10:128. 10.1186/1472-6963-10-128 20478043 PMC2893175

[B34] WHO Regional Office for Europe (2015). Access to New Medicines in Europe: Technical Review of Policy Initiatives and Opportunities for Collaboration and Research. Copenhagen: World Health Organization; Regional Office for Europe.

